# Case Report: A Case Series of Immunobiological Therapy (Anti-TNF-α) for Patients With Erythema Nodosum Leprosum

**DOI:** 10.3389/fmed.2022.879527

**Published:** 2022-06-24

**Authors:** Ana Flávia Moura Mendes, Ciro Martins Gomes, Patrícia Shu Kurizky, Mayra Ianhez

**Affiliations:** ^1^Dermatologia, Hospital de Doenças Tropicais, Goiânia, Brazil; ^2^Departamento de Dermatologia, Universidade de Brasília (UnB), Brasília, Brazil; ^3^Dermatologia, Universidade Federal de Goiás (UFG), Goiânia, Brazil

**Keywords:** leprosy, dermatology, immunosuppression therapy, autoimmunity, leprosy reaction

## Abstract

Patients with leprosy may experience a chronic and severe type II leprosy reaction (ENL) erythema nodosum leprosum that may not respond to thalidomide and systemic immunosuppressants or may even cause serious adverse events. We here present four patients in whom anti-TNF-α therapy was used with successful results and compare our findings with other published cases. Four patients with chronic and severe ENL who did not respond to, at least, thalidomide and steroids (high doses) were followed up at two reference centers in Brazil. A thorough laboratory investigation was performed to exclude tuberculosis and other diseases before the start of immunobiological medication. Three patients were started on etanercept, and one patient was started on adalimumab. Of all patients, three developed severe adverse events resulting from the use of classical immunosuppressants for ENL (cataracts, deep vein thrombosis, diabetes, and osteoporosis). In all cases, a reduction in the number of ENL and, at least half of the immunosuppressant dose between 6 months and 2 years, were observed. Long-term follow-up of one patient revealed a dramatic reduction in hospital admissions due to ENL, from 12 instances in 1 year (before biologic therapy) to none (after biologic therapy), along with an improvement in condyloma acuminatum. In addition, no direct adverse events were observed with biologics. Treatment with anti-TNF-α therapy may be used as an alternative in patients with chronic and severe ENL who do not respond to traditional treatment (e.g., thalidomide, steroids, and other immunosuppressants). This treatment can help reduce the frequency of ENL, the immunosuppressive burden, and the number of hospital admissions.

## Introduction

Leprosy is a disabling disorder that mainly affects the skin and the peripheral nervous system. This disorder may be complicated by immune-mediated inflammatory reactions, such as reversal reaction (type I reaction) and erythema nodosum leprosum (ENL, type II reaction), as well as by peripheral nerve damage (1, 2).

Erythema nodosum leprosum is a clinical feature of multiple systemic symptoms, such as fever, crops of tender erythematous nodules, neuritis, arthritis, orchitis, lymphadenitis, and iritis (1, 2). It is considered an emergency event (3), which may affect 5–10% of patients with borderline leprosy and up to 50% of those with lepromatous leprosy (LL) (4). Given its magnitude and social significance, including the possibility of social stigma and sequelae for the patients and their families, leprosy is still one of the most serious public health problems in Brazil (5).

Among the most common treatments prescribed for ENL are thalidomide, prednisone/prednisolone (systemic steroids), and clofazimine (3). However, using high doses of steroids for prolonged periods of time may lead to well-known side effects, such as diabetes, cataracts, osteoporosis, and obesity (2, 6). Similarly, thalidomide has highly teratogenic effects and must be used with extreme caution, especially for women at childbearing age (2, 3). Both thalidomide and systemic steroids are associated with a risk for deep vein thrombosis and pulmonary embolism, and they even have a synergic risk effect if used in combination (3). Clofazimine is associated with gastrointestinal effects and skin pigmentation and may pose a cosmetic issue (7). Furthermore, systemic immunosuppressants, such as methotrexate, azathioprine, and cyclophosphamide, may increase the risk of infection and hepatic and renal damage (2, 8).

Although thalidomide is the first-line treatment for ENL in many countries, combining it with at least one additional agent, such as pentoxifylline or an immunosuppressant (3, 8), may be necessary for refractory cases (9). Multibacillary patients may experience ENL for many months (3, 8). Therefore, not only can ENL expose its patients to different sequelae and neurological impairments, but also the therapies targeting it may pose a high risk of side effects (7).

Several alternative therapies have been proposed for ENL, such as vaccination with *Mycobacterium indicus pranii* (10), apremilast (an oral phosphodiesterase IV inhibitor), cyclosporine, and tenidap (an anti-inflammatory drug) (7), as well as anti-TNF-α therapy (1, 2, 6, 11, 12). We here present four patients with severe, recalcitrant, and chronic ENL who did not respond to multiple therapeutic regimens and were treated with anti-TNF-α therapy. We compared these cases to other similar cases and discussed the rationale behind the use of anti-TNF-α therapy.

## Case Description

All four patients presented with anesthetic skin patches and/or thickened nerves and acid-fast bacilli on slit skin smears and/or a histopathology compatible with leprosy or ENL (9). Leprosy was classified according to the Ridley–Jopling system using clinical, histological, and bacteriological indices (13). A case definition of ENL was considered if a patient presented with crops of tender cutaneous or subcutaneous erythematous nodules, with or without systemic symptoms, or a suggestive histopathology (9).

The nature of ENL was defined as acute for a single episode lasting less than 24 weeks, recurrent for a patient experiencing a second episode of ENL 28 days or more after stopping the treatment for ENL, and chronic if over 24 weeks or more a patient required ENL treatment either continuously or 27 days or less after the last treatment period (14). In terms of severity, ENL can be classified according to the clinical manifestations as mild if the patient presented with less than 10 nodules per affected body, moderate if the patient presented with 10–20 nodules, and severe if the patient presented with more than 20 nodules (15). This study was approved by the institutional review boards of the original centers (CAAE: 93279318.9.0000.5558), and written consent was obtained from each individual.

Table 1 shows the clinical data of the four patients, along with another five similar published cases. As can be observed, the ratio between the males and females was equal (1:1), with a median age of 44.2 [range: 29–59] years for patients with ENL. The patients were originally from two hospitals at the center of Brazil: Hospital de Doenças Tropicais (Goiás) and Hospital Universitário de Brasília (Distrito Federal). All four patients were diagnosed with LL, according to the Ridley–Jopling classification (13). At the time of diagnosis, they presented with a bacteriological index (BI) of five or more on at least four body sites, and their ENL began when they were started on multidrug therapy (MDT). One of the patients was treated twice with MDT. ENL was classified as severe and chronic with common morphological features compatible with multiple (>50) tender, erythematous papules and nodules, followed by systemic symptoms, such as fever, malaise, arthralgia, joint swelling, and peripheral edema with neurological features of neuritis. No reversal reaction was observed in any of the patients. Thalidomide and high doses of systemic steroids were used in all patients. Methotrexate was used in two patients (Patients 1 and 3). However, in Patient 1, the fourth drug was pentoxifylline. These drugs were used for at least 1 year. Patient 1 was started on high doses of thalidomide, systemic steroids, methotrexate, and pentoxifylline for 4 years. Three patients developed severe adverse events because of the ENL treatment (Patients 1, 2, and 4), including cataracts, deep vein thrombosis (Patient 4), diabetes, obesity, and osteoporosis.

**Table 1:** Demographic and clinical data of leprosy patients with severe, chronic and refractory ENL (*n* = 4 patients), and cases already published (*n* = 5 patients) (2006–2021).

	**Patient 1**	**Patient 2**	**Patient 3**	**Patient 4**	**Patient 5** **Faber et al. (2006)**	**Patient 6** **Ramien et al. 2011**	**Patient 7** **Chowdhry et al. 2016**	**Patient 8** **Santos et al. 2017**	**Patient 9** **Cogen et al. (2020)**
Sex	F	M	F	M	F	F	M	M	M
Age in years (at diagnosis)	29	32	57	59	52	33	49	40	28
Country	Brazil	Brazil	Brazil	Brazil	Argentina	Philipines	India	Brazil	Samoa
Leprosy classification	LL	LL	LL	LL	BL	LL	LL	LL	LL
BI at leprosy diagnosis	5	5.25	6	5.5	5	2–4 (at 6 sites)	6	NI	5
Temporal relationship of reactional status and MDT	6 months after MDT	During MDT treatment	During MDT treatment	During MDT treatment	1.5 years after MDT	Mild ENL was present prior to MDT	2 months after MDT	Since the diagnosis of leprosy	During MDT treatment
Number of MDT treatment	2	1	1	1	1	2-year treatment	NI	1	MDT and change after to clofazimine + levofloxacin + clarithromycin
ENL type	Chronic	Chronic	Chronic	Chronic	Chronic	Chronic	Chronic	Chronic	Chronic
ENL severity	Severe	Severe	Severe	Severe	NI	NI	Severe	NI	NI
Morphologic features of ENL	Erythematous nodules, necrotic, and ulcerated lesions	Erythematous nodules, necrotic, and ulcerated lesions	Painful erythematous nodules and plaques	Painful erythematous nodules and plaques	Painful erythematous nodules and plaques	Erythematous papules and nodules	Erythematous tender papules, nodules and necrotic ulcers	Nodules	Painful and ulcerated cutaneous nodules
Symptoms related to ENL	Fever, malaise, arthalgia, joint swelling, peripheral oedema	Fever, malaise, arthalgia, joint swelling, peripheral oedema	Fever, malaise, arthalgia, joint swelling, peripheral oedema	Fever, malaise, arthalgia, joint swelling, peripheral oedema	Tender axillary lymph node	Fever, malaise	High grade fever, myalgia, lymphadenopathy and epididymo-orchitis	NI	Polyarthitis, acral oedema, and uveitis
Neurologic impairment	Neuritis, neuropathy	Diffuse neuritis	Diffuse neuritis	Diffuse neuritis	Thickened and tender ulnar nerve	Neuropathy	Severe neuritis	NI	Peripheral neuritis
Reversal reaction	No	No	No	No	NI	NI	NI	NI	NI
Skin biopsy	No	Consistent with ENL	Consistent with ENL	Consistent with ENL	NI	NI	Consistent with ENL	NI	NI
Number of ENL at the time of anti-TNF	50	>50	>50	>50	NI	NI	NI	NI	NI
Therapy for ENL prior to anti-TNF-α therapy	Thalidomide 400 mg/day, prednisone 1.5 mg/kg/day, methotrexate 15 mg/week, pentoxifylline 1,200 mg/day	Thalidomide 200 mg/day, prednisone 1 mg/kg/day	Thalidomide 400 mg/day, prednisone 1 mg/kg/day, methotrexate 20 mg/week	Thalidomide 200 mg/day, prednisone 1 mg/kg/day	Thalidomide 300 mg/day, prednisolone 40 mg/day, pentoxifyline 1,200 mg/day	Thalidomide 100 mg/day, prednisone 80 mg/day, clofazimine	Thalidomide 300 mg/day, prednisolone up to 80 mg/day, clofazimine 300 mg/day, clarithromycin 1 g/day, ofloxacin 400 mg/day, pentoxifylline 1,200 mg/day, azathioprine 300 mg/day	Thalidomide 300 mg/day and Prednisone up to 40 mg/day	Thalidomide 300 mg/day, prednisone up to 120 mg/day, clofazimine 150 mg/day, cyclophosphamide 200 mg/day, prednisolone eye drops
Time between using ENL treatment and anti- TNF-α therapy	4 years	1 year	2 years	1 year	NI	6 years	6 months	4 years	NI
Adverse events related to classical ENL treatment	Osteoporosis, cataracts, giant condyloma acuminatum, insulin resistance, 12 hospitalizations	Obesity, diabetes	None	Cataracts, arterial thrombosis	NI	Cushingoid features, hypertension, osteoporosis, toe fractures, recurrent soft-tissue infections, deep-vein trhombosis	Oral candidiasis, abdominal pain, diarrhea, worsening of his diabetes, hypertriglyceridemia and aggravation of peripheral neuropathy with thalidomide	NI	3 hospitalizations
Anti-TNF-α therapy	Etanercept 50 mg/week SC	Adalimumab 40 mg/biweekly SC	Etanercept 50 mg/week SC	Etanercept 50 mg/week SC	Infliximab 5 mg/kg	Etanercept 50 mg/week	Etanercept 50 mg/week for 16 weeks and then biweekly for 16 weeks	Etanercept 50 mg/week	Infliximab 5 mg/kg in 7 infusions —weeks 0, 2, 7, 16, 78, and 88, 99
Time for symptoms to subside after anti-TNF- α therapy	3 days	1 week	1 month	1 month	Hours	6 weeks	48 h in the first dose	48 h experiencing recurrence after 7 days	Immediate
Duration of anti-TNF- α therapy	1.5 years	6 months	1 year	2 years	Infusion in weeks 0.2 and 6	2 years and remain asymptomatic 2.5 years after anti-TNF-α stop	32 weeks	11 months	7 infusions when flares
ENL treatment after anti-TNF -α therapy	Thalidomide 100 mg/day and prednisone 0.5 mg/kg/day (1 year after anti-TNF therapy)	Thalidomide 100 mg/day and prednisone 0.5 mg/kg/day	Thalidomide 100 mg/day and prednisone 20 mg/day	Prednisone 0.5 mg/kg/day	No treatment after 1 year	After year, just thalidomide 100 mg/day	Prednisolone discontinuation after 12 weeks	Prednisone 10 mg/week	Discontinuation of prednisone after 4 years
Adverse events related to anti-TNF-α therapy	No	Sepsis (during the concomitant use of adalimumab and prednisone 1 mg/Kg/day)	No	No	NI	NI	NI	NI	7th infusion: hypotension, oedema, and throat tightening

*F, female; M, male; LL, lepromatous leprosy; BI, baciloscopic index; MDT, multidrug therapy; ENL, erythema nodosum leprosum; SC, subcutaneous; TNF, tumoral necrosis factor; NI, not informed*.

**Figure 1:**
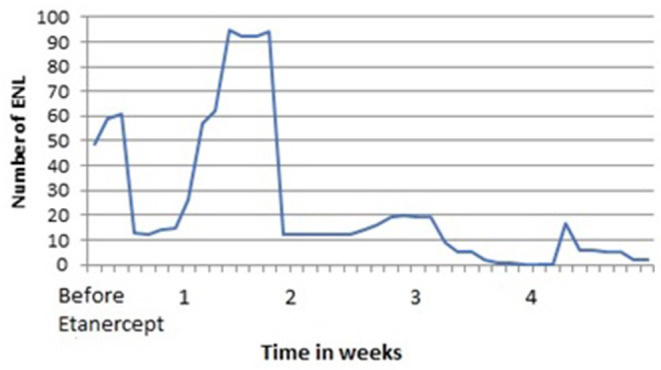
Behavior of the reduction of ENL after etanercept therapy in Patient 1.

Before anti-TNF-α therapy was started, a complete investigation was performed to rule out latent foci of tuberculosis (Mantoux test and chest X-ray), hepatitis B and C, and HIV. Etanercept was the preferred anti-TNF-α therapy in three patients, with one patient undergoing therapy using adalimumab. Improvements were observed as early as 3 days (Figure 1) to 1 month, and the duration of anti-TNF-α therapy varied from 6 months to 2 years.

The outcomes considered to judge an improvement as a result of anti-TNF-α therapy were as follows: a reduction in the number of ENL cases, a reduction in the rate of hospitalization, and a reduction in the dosage of common treatments for ENL (thalidomide, steroids, methotrexate, pentoxifylline, or clofazimine). All patients exhibited a reduction in ENL, which particularly manifested as a reduction in the number of ENL, in weeks, in Patient 1 (Figure 1). Interestingly, in the year before the anti-TNF-α therapy, Patient 1 was hospitalized 12 times for ENL. However, in the year after therapy, she was hospitalized only once for neuritis. She presented with genital condyloma acuminatum, which was resistant to different treatment modalities. However, after methotrexate was stopped and the dose of systemic steroids was decreased, she improved without a specific treatment for human papillomavirus. Overall, a reduction of at least half in thalidomide and systemic steroids was observed in all four patients on anti-TNF-α therapy over a duration of 6 months−2 years.

## Discussion

Leprosy is a severe, neglected chronic disease. Besides the physical disabilities resulting from the neurological sequelae of this disease, type II reactional states are considered a public health problem in Brazil (12).

Our cases did not demonstrate a gender-related difference for ENL. In fact, several studies have highlighted that age and gender are not risk factors for ENL (6). Rather, the risk factors for developing ENL include a LL type and a high bacillary index. The relative risk of developing ENL is 3.6 with an LL spectrum and 8.6 with a bacillary index of 6, which is the case for patients with refractory, chronic, and severe ENL (16). Although the onset of ENL has been found to be highest during the first year of MDT (17), some studies claim a higher incidence in the second and third years after MDT is started (7). In three patients, MDT was changed (Patient 9) or repeated (Patients 1 and 6). Although the World Health Organization has estimated the rate of relapse after MDT as 0.77% for multibacillary patients 9 years after treatment (18), ENL may still pose a significant challenge to differentiate from a recurrence of leprosy, especially after many years of MDT (3).

Neurological impairments are common in patients with ENL (8, 19). Recently, Andrade et al. (19) described a link between demyelination and acute neuritis during leprosy. However, it remains unknown whether demyelination is a consequence of the inflammatory process of neuritis or whether it is directly induced by *Mycobacterium leprae*. Persistent demyelination is associated with axonal damage, which progressively compromises large fibers, leading to motor impairment. Damage to the myelin sheath may be a consequence of the inflammatory process resulting either from humoral immunity or from the release of immune mediators. TNF acts on Schwann cells (SCs) by stimulating the production of IL-6 and IL-8, contributing both directly and indirectly to neuroinflammation (20). Moreover, *M. leprae* stimulate the secretion of IL-23 in SCs, a cytokine that is believed to be involved in demyelinating processes. Thus, focal demyelination may potentially become a prime target for therapeutic interventions aimed at improving nerve function during leprosy (19). One of the contraindications for the use of anti-TNF-α therapy is demyelinating disorders (21). Since demyelination is regarded as a new discovery in leprosy (19), carefully evaluating patients who undergo this treatment to control their ENL is important. Importantly, none of our cases showed any neurological impairments caused by anti-TNF-α therapy.

We compared all cases with already published ones and found that most of the patients who were started on anti-TNF-α therapy were using at least two medications at high doses for 6 months−6 years (Table 1). Although thalidomide is regarded as the drug of choice for treating ENL, using it at high doses for prolonged periods of time may cause nerve injury and be a confounding factor with the neurological effects of the disease itself (1). Prednisone is added to the treatment regimen when the reactions are difficult to control or in the presence of complications, especially neuritis (22). The data outlined in Table 1 show that prolonged classical treatment is associated with increased side effects (Patients 1, 7, and 8) (1, 12). In addition, the synergic association between systemic steroids and high doses of thalidomide is considered a risk factor for deep vein thrombosis (Patients 4 and 6) and pulmonary thromboembolism (6). Hence, precociously using anti-TNF-α therapy may prevent prolonged exposure to immunosuppressant agents and their side effects (2).

Etanercept was the most used drug for ENL, followed by infliximab and adalimumab. Infliximab is a human-murine chimeric monoclonal antibody against TNF-α, and etanercept is a dimeric fusion protein of the extracellular portion of the p75 TNF receptor coupled to IgG1. Both agents can effectively reduce the levels of TNF-α (7). Although the underlying immunological mechanism of ENL remains unclear, it is important to highlight the role of cytokines. High levels of TNF-α and IL-6 are consistently found in patients with severe disease (23). Moreover, the overexpression of TNF-α and the fact that thalidomide is an important anti-TNF-α agent allowed Farber et al. (11) to treat a patient with an uncontrolled type II reaction using classic systemic therapy with infliximab. After this study, four more cases were described, but only one patient continued to use infliximab (2). Etanercept was chosen instead of infliximab because of the increased risk of reactivation of latent tuberculosis and granulomatous diseases (24). The disadvantage of infliximab is its mode of administration (endovenous), which requires a hospital setting and may lead to adverse events, such as hypotension, edema, and throat tightness (2). Only one study has focused on adalimumab in the context of the reversal reaction, but not in that of the type II reaction. Compared to the administration of prednisolone for 20 weeks, the efficacies of both drugs against skin lesions in the context of the reversal reaction were similar. Interestingly, adalimumab is superior to prednisolone in terms of improving the nerve function and sensory and motor loss (25).

Unlike psoriasis, which is also treated using anti-TNF-α therapy, reactive leprosy episodes seem to exhibit a rapid response. The improvement observed in ENL seems to be immediate or within hours with infliximab (Patients 5 and 9) (2, 11), whereas etanercept may take days to a month (Patients 1, 2, 3, 4, 6, 7, and 8) (1, 6, 12). However, since only nine patients underwent anti-TNF-α therapy for ENL, we cannot guarantee the superiority of a specific anti-TNF-α agent. Nevertheless, in psoriasis, another skin disease treated by this class of drugs, infliximab (26) and adalimumab (27) have a faster response and are more efficacious than etanercept, in achieving a clear or almost clear stage.

The dosage of anti-TNF-α therapy used for treating ENL is considered another challenge. Should biologics be administered only during flares or perhaps as a prophylactic therapy? In Patients 5, 7, and 9, the drug was administered at a custom dose, preferably during flares. Since ENL flares decrease over time, specific drugs for ENL treatment are expected to be tapered down (7). However, as systemic steroids are the most detrimental drugs for such patients, it is advisable to stop such drugs in advance and to continue anti-TNF-α therapy along with thalidomide. It is also worth noting that the gaps between the doses of anti-TNF-α therapy may lead to the formation of neutralizing antibodies and, hence, the loss of efficacy (28). Therefore, maintaining a uniform and specific dose is considered a favorable choice.

Overall, no side effects with anti-TNF-α therapy were observed, except in Patient 2, who had sepsis but has also been on systemic steroids for a year. During the early 2000s, when anti-TNF-α therapy was released, the side effects of this type of therapy were strongly debated. In addition, the well-established connection between the inhibition of TNF-α and reactivation of tuberculosis may similarly hold true for leprosy. The rapidity with which patients develop signs of leprosy after receiving the biological agent suggests possible recrudescence of latent leprosy or initial misdiagnosis. Therefore, using anti-TNF-α therapy for patients with leprosy before complete treatment with MDT may reduce the efficacy of antimicrobial agents or promote infection by *M. leprae*. Although anti-TNF-α therapy is contraindicated in the case of lupus and cardiac congestive failure (stages III–IV) (29), given their target specificity, anti-TNF agents are considered safer than a wide immunological blockade provided by steroids and other immunosuppressants (30, 31).

The indications of anti-TNF-α agents are increasing in many diseases (21). Despite their high cost, more affordable biosimilars are reaching the market (2). It is also worth highlighting that the indication of biologic therapy for ENL may be limited because ENL occurs in ~50% of LL cases (2). Moreover, flares are auto-limited, especially within the first 3 years after MDT, and only 26.8% of patients with ENL require at least one additional agent (16).

Our study has some limitations. For example, it lacks prospective controls (as in all case reports), it lacks laboratory tests aimed at verifying the behavior of anti-inflammatory markers, and it lacks more well-defined and uniform outcome measures for ENL. Hence, some questions remain unanswered: Who is the best patient with ENL for biologic therapy? Is it safe to use biologic therapy along with MDT or is it necessary to wait until treatment is completed? For how long should systemic agents be used until classical treatment of ENL is considered a failure? What is the best anti-TNF-α candidate to treat ENL? What is the best strategy, during flares or as a prophylactic? How does anti-TNF-α therapy affect nerve damage?

In our series, patients who presented with recalcitrant, chronic, and severe ENL were classified as LL patients with a BI higher than five, who had their first episode of ENL within the first year of MDT along with neurological impairments. All patients who received biologic therapy did not respond to high-dose systemic steroids or thalidomide. Anti-TNF-α therapy demonstrates rapid improvements and allows the reduction of ENL, the frequency of hospitalizations, and the doses used of classical drugs. Clinical trials on this population are, therefore, important to determine the role of biologicals in leprosy and neurological impairments and their reactive states.

## Data Availability Statement

The original contributions presented in the study are included in the article/supplementary material, further inquiries can be directed to the corresponding author/s.

## Ethics Statement

The studies involving human participants were reviewed and approved by Comite de Etica do Hospital de Doenças Tropicais - HDT GO and by the Comitê de Ética da Faculdade de Medicina da Universidade de Brasília. The patients/participants provided their written informed consent to participate in this study. Written informed consent was obtained from the individual(s) for the publication of any potentially identifiable images or data included in this article.

## Author Contributions

AM: formal analysis, investigation, and resources. CG, PK, and MI: supervision, validation, visualization, writing—original draft, and writing—review and editing. All authors contributed to the article and approved the submitted version.

## Funding

This study was financed in part by Fundo de Apoio à Dermatologia (FUNADERM)—Sociedade Brasilleira de Dermatologia (SBD).

## Conflict of Interest

The authors declare that the research was conducted in the absence of any commercial or financial relationships that could be construed as a potential conflict of interest.

## Publisher's Note

All claims expressed in this article are solely those of the authors and do not necessarily represent those of their affiliated organizations, or those of the publisher, the editors and the reviewers. Any product that may be evaluated in this article, or claim that may be made by its manufacturer, is not guaranteed or endorsed by the publisher.
